# Do prisoners trust the healthcare system?

**DOI:** 10.1186/s40352-021-00141-x

**Published:** 2021-07-03

**Authors:** Lindsey A. Vandergrift, Paul P. Christopher

**Affiliations:** 1 Warren Alpert Medical School, Biomedical Department, Brown University, 69 Brown St G-9474, RI 02903 Providence, USA; 2 Department of Psychiatry and Human Behavior , Brown University , RI 02912 Providence, USA

**Keywords:** Healthcare, Distrust, Criminal justice, Incarceration

## Abstract

**Background:**

Individuals who are incarcerated have greater healthcare needs than non-justice-involved individuals, yet incarcerated individuals often report substandard care. There are disproportionate numbers of black, indigenous, and people of color (BIPOC) in prison, who, even in general society face greater obstacles to accessing healthcare and have worse health outcomes due to structural racism. Regardless of race, people with criminal justice involvement often report stigma from the non-carceral healthcare system. Providing sufficient healthcare in carceral settings themselves is complicated by lack of privacy and the inherent dialectic of prisons that restrict freedom and providers focusing on healing and health. Based on these adverse experiences, people who are incarcerated may have decreased distrust in the healthcare system, deterring individuals from getting adequate medical care.

**Methods:**

In this exploratory study, health care system distrust was evaluated among 200 people who were incarcerated using the Revised Health Care System Distrust scale, a community-validated, 9-item measure comprised of 2 subscales (*values* and *competence* distrust).

**Results:**

Distrust was moderately and positively associated with participant age (*r*_*s*_ = 0.150, *p* = 0.034), with the second-oldest quintile (33 to 42-year-olds) reporting the highest level of overall and competence distrust. Participants identifying as Non-Latinx White reported higher competence distrust compared to Latinx and Non-Latinx/Non-White respondents.

**Conclusions:**

These preliminary findings suggest that select groups of prisoners may be less likely to trust the healthcare system, highlighting an impediment to receiving adequate care while incarcerated. Further study of this topic is warranted.

**Supplementary Information:**

The online version contains supplementary material available at 10.1186/s40352-021-00141-x.

## Background

People experiencing incarceration shoulder a disproportionate burden of severe illnesses (Harlow, 1999; James & Glaze, 2006; Karberg & James, 2005; Maruschak & Beavers, 2009; Mumola & Karberg, 2006; Steadman, Osher, Robbins, Case, & Samuels, 2009) and have shorter life expectancy compared to those who have never been incarcerated (Patterson, 2013). Across various criminal justice settings, individuals experience higher rates of past trauma or abuse (Harlow, 1999), chronic illness (James & Glaze, 2006), severe mental illness (Steadman et al., 2009), substance abuse (Karberg & James, 2005; Mumola & Karberg, 2006), and infectious diseases (Maruschak & Beavers, 2009; Massoglia, 2008). The stress imposed by incarceration itself further contributes to negative health outcomes (McEwen & Stellar, 1993). Despite these myriad health-related challenges, individuals who are incarcerated often report sub-standard care (Christopher et al., 2017).

The poor healthcare and health status of incarcerated individuals is further complicated by the disproportionately high representation of black, indigenous and people of color (BIPOC) in prison, who at baseline face greater obstacles to accessing healthcare and have worse health outcomes due to structural racism. The effect of structural racism on health includes worse health outcomes for Blacks in almost all categories, such as infant mortality, life expectancy, and self-assessed health status (National Center for Health Statistics. Health, United States, 2015, 2016). Inequitable criminal laws and policing practices, such as the “war on drugs” that have implicitly targeted BIPOC individuals and led to higher conviction rates of Blacks for drug possession (Ghandnoosh, 2015), also exacerbate these disparities by shunting people of color into jails and prisons rather than treatment, where the cycle of poor health continues (Bailey et al., 2017; Institute of Medicine Committee on U, Eliminating R, Ethnic Disparities in Health C, et al., 2003). Moreover, the inequitable distribution of (and access to) other societal resources and goods that promote individual health (i.e., housing, education, employment, earnings, benefits, credit, media, and so on) further increase the risk for adverse health outcomes among BIPOC persons (Bailey et al., 2017).

Impediments to providing sufficient healthcare in prison include the complex health problems that prisoners face and often inadequate financial resources allocated by correctional systems to properly treat these conditions (Ditton, 1999; Harlow, 1999; James & Glaze, 2006; Karberg & James, 2005; Maruschak & Beavers, 2009; Mumola & Karberg, 2006; Steadman et al., 2009). Moreover, the correctional system is primarily concerned with punishment, rehabilitation, and public safety; these priorities may compete with, if not obstruct, the aims of the healthcare system, which are to improve health and well-being through a foundation of trust and mutual respect between patient and provider (Wilper et al., 2009). Indeed, specific policies in some correctional settings directly disrupt medical care by requiring that correctional officers be present during medical interviews and exams, requiring that individuals pay the equivalent of 25 h of labor to schedule an appointment, and by constraining available treatment options through prison regulations and drug formularies (Ghandnoosh, 2015).

Collectively, these institutional conditions may create a mindset of distrust, contributing to prisoners’ reluctance to seek care. There are multiple reasons why people who are incarcerated may distrust the healthcare system. First, people in the correctional system often perceive the quality of correctional healthcare as inferior to that available in the community and perceive the interpersonal treatment by correctional healthcare workers as less humane (Christopher et al., 2017). Despite reporting a more positive perception of healthcare workers in non-carceral settings, individuals with a history of criminal justice system involvement nevertheless often experience discrimination from these community providers, which can adversely impact trust and healthcare utilization (Frank, Wang, Nunez-Smith, Lee, & Comfort, 2014). Discouraging people from seeking care, even if through unintentional discrimination, may also worsen distrust and cause people to disengage from the healthcare system altogether.

Distrust in the healthcare system is sometimes cited to explain why certain individuals experience worse health outcomes, claiming, for example, that non-incarcerated individuals are less willing to seek care (LaVeist, Isaac, & Williams, 2009; Musa, Schulz, Harris, Silverman, & Thomas, 2009; Whetten et al., 2006; Yang, Matthews, & Anderson, 2013; Yang, Matthews, & Hillemeier, 2011) or comply with medications (Dean, Moss, McCarthy, & Armstrong, 2017). While such associations may be factually true, the implication is often that individuals are to blame for their poor health outcomes. Such characterizations obfuscate the complicated and pernicious effects of the structural inequities that undermine healthcare access and health more broadly. Instead, it may be more accurate to view healthcare distrust as a downstream effect of chronic exposure to these inequities. Seen in this light, distrust may also serve as a marker to identify individuals and communities in greatest need of better policies and practices to address gaps in care (Boyd, Lindo, Weeks, & McLemore, 2020).

At present, little is known about the extent to which prisoners trust the healthcare system. Given the potential for healthcare distrust among prisoners and the likelihood that distrust would exacerbate existing obstacles to addressing their need for medical care while incarcerated, this is an important topic of inquiry. The present exploratory study seeks to address this knowledge gap using a 9-item scale of Health Care System Distrust (validated in the general population) (Boyd et al., 2020) to examine overall healthcare system distrust among male and female individuals who are incarcerated and its association with select demographic factors.

## Methods

### Participants

Participants were recruited from two clinical trials being conducted within a single-state Department of Corrections (DOC) for both sexes: one study was testing a safety planning intervention for incarcerated individuals with suicidal ideation, and the other was a study testing a service linkage intervention for incarcerated individuals with alcohol use disorder. Between July 2016 and July 2017, research staff from these two clinical trials informed prospective study participants of the opportunity to learn about the present add-on study. Individuals who expressed interest were subsequently contacted by a separate research team member of this study and engaged in an informed consent discussion. All participants were told that their participation decision would not impact their total incarceration time; that all responses would be kept confidential; and that no information provided during the study would be shared with research staff of the clinical trials from which they were recruited, DOC staff, court or parole officers, or anyone else involved in the correctional setting. Under instruction from the approving institutional review board, no information was provided from the two clinical trials on the number of individuals who declined to be contacted for the present study. Among those who expressed interest in this study, all chose to participate and provided verbal and written informed consent and were compensated $10 for their participation in accordance with DOC policy. The institutional review board of [omitted for peer review] approved this study.

### Measures

Participants completed the Revised Health Care System Distrust scale (Shea et al., 2008), a measure which asks respondents to rate their level of agreement from 1 to 5 for nine items that address different aspects of trust in the healthcare system (Table 2) with possible summative scores ranging from 9 to 45. The measure assesses two domains, a 5-item subscale examining values distrust (e.g., “The health care system puts making money above patient needs”) and a 4-item subscale examining competence distrust (e.g., “The health care system makes too many mistakes”); maximum scores for the values and competence subscales are 25 and 20, respectively. The scale, which was developed and validated for use in a general population, showed strong reliability in this study sample: Crohnbach’s *α* = 0.881 across all nine items, *α* = 0.819 for values distrust items, and *α* = 0.788 for competence distrust items. Participants also provided the following demographic information: age, sex, self-identified race and ethnicity, and education. Race and ethnicity were coded into three representative groups for analyses while still capturing experiences of two large BIPOC subgroups. Age was analyzed as a continuous variable and as evenly-divided quintiles.

### Statistical analysis

Data analysis was performed using R 3.6.0. Descriptive statistics were used to summarize the sample population. Spearman rank correlation was performed between distrust score and age. The continuous measurement of distrust was compared against categorical variables (age quintiles, race/ethnicity, sex, and schooling level) using ANOVA and subsequent pairwise analysis for multi-item categories (age quintiles, race/ethnicity, and schooling level) with alpha *p* < 0.05 and no adjustment for multiple comparisons.

## Results

### Demographics

The study population contained 137 males (68.5%). Mean age was 32.7 ± 10.9 years. Forty-seven (23.5%) individuals identified as Latinx, 99 (49.5%) as Non-Latinx White, and 54 (27%) as Non-Latinx/Non-White. Fifty-two individuals (26.1%) reported some high school education, 82 (41.2%) had graduated high school or earned a GED, and 40 (20.1%) had some college education. Additional information is provided in Table 1.

**Table 1:** Study Population Characteristics and Responses to Distrust of Health Care System Scale

	n (%)	Distrust of Health Care System Score (range 9 to 45)		Values Distrust (range 5–25)		Competence Distrust (range 4–20)	
		*Mean (95% CI)*	*p value*^*a*^	*Mean (95% CI)*	*p value*^*a*^	*Mean (95% CI)*	*p value*^*a*^
**Age**							
19–23	40 (20)	23.0 (20.8–25.1)	0.028	12.9 (11.6–14.2)	0.147	10.1 (9.1–11.1)	0.013
24–27	40 (20)	21.3 (19.2–23.5)		11.7 (10.4–13.0)		9.7 (8.7–10.7)	
28–32	40 (20)	23.1 (20.9–25.2)		13.1 (11.7–14.4)		9.9 (8.9–10.9)	
33–42	40 (20)	26.3 (24.2–28.5)^b^		14.1 (12.7–15.4)		11.9 (11.0–12.9)^c^	
43–66	40 (20)	23.6 (21.5–25.7)		12.4 (11.0–13.7)		10.8 (9.8–11.8)	
**Race/Ethnicity**							
Non-Latinx White (NLW)	99 (49.5)	24.0 (22.7–25.4)	0.090	12.8 (12.0–13.7)	0.179	10.9 (10.3–11.6)	0.046
Non-Latinx/Non-White	54 (27)	21.7 (19.8–23.5)		12.1 (10.9–13.2)		9.6 (8.7–10.4)^d^	
Latinx	47 (23.5)	24.2 (22.2–26.2)		13.6 (12.4–14.9)		10.6 (9.7–11.5)	
**Sex**							
Male	137 (68.5)	23.6 (22.4–24.8)	0.661	12.9 (12.2–13.7)	0.466	10.5 (9.9–11.0)	0.960
Female	63 (31.5)	23.1 (21.4–24.8)		12.5 (11.4–13.5)		10.5 (9.7–11.3)	
**Schooling**							
8th grade or less	10 (5.0)	23.2 (18.9–27.5)	0.429	12.1 (9.5–14.7)	0.296	10.7 (8.7–12.7)	0.555
Some HS	52 (26.1)	23.7 (21.8–25.6)		12.8 (11.6–13.9)		10.7 (9.8–11.6)	
HS graduate or GED	82 (41.2)	22.5 (21.0–24.0)		12.3 (11.4–13.2)		10.0 (9.3–10.7)	
Some college	40 (20.1)	24.9 (22.7–27.1)		14.2 (12.9–15.5)		10.9 (9.9–11.9)	
College graduate	10 (5.0)	23.5 (19.2–27.8)		12.4 (9.8–15.0)		10.5 (8.5–12.5)	
Beyond college	5 (2.5)	27.2 (21.1–33.3)		13.8 (10.1–17.5)		12.2 (9.3–15.1)	

^a^group ANOVA *p* values

^b^pairwise ANOVA comparison between quintile 4 and quintiles 1, 2, and 3, respectively (*p* < 0.04 for all)

^c^pairwise ANOVA comparison between quintile 4 and quintiles 1, 2, and 3, respectively (*p* < 0.01 for all)

^d^pairwise ANOVA comparison between Non-Latinx/Non-White and Non-Latinx White (*p* < 0.02)

**Table 2:** Health Care System Distrust Scale Questions and Subscales. ^a^Item is reverse scored

	Competence / Value Subscale
1. The health care system does its best to make patients’ health better.^a^	C
2. The health care system covers up its mistakes.	V
3. Patients receive high-quality medical care from the health care system.^a^	C
4. The health care system makes too many mistakes.	C
5. The health care system puts making money above patients’ needs.	V
6. The health care system gives excellent medical care.^a^	C
7. Patients get the same medical treatment from the health care system, no matter what the patient’s race or ethnicity.^a^	V
8. The health care system lies to make money.	V
9. The health care system experiments on patients without them knowing.	V

### Distrust and age

Older participants were significantly and moderately more likely to express distrust (*r*_*s*_ = 0.150, *p* = 0.034). Competence-related distrust was the primary driver of this association (*r*_*s*_ = 0.186, *p* = 0.009).

ANOVA analysis of quintile-divided age groups showed that the second-oldest group (33 to 42 years) expressed the highest level of distrust (Table 1, *p* = 0.028). In pairwise analyses, the 33 to 42-year-old group endorsed the highest overall distrust and the highest competence distrust compared to the lowest three quintiles (Table 1). The 33 to 42-year-old group showed significantly higher distrust compared to the 43 to 66-year-old group (as well as all other groups) for two items (Supplementary Table 1, Items 3 and 8). All item-level comparisons are shown in Supplementary Table 1.

### Distrust and race/ethnicity

Non-Latinx White participants reported significantly higher competence distrust than the Non-Latinx/Non-White group but not significantly greater than the Latinx group (Table 1). No significant differences in overall distrust were found between racial/ethnic groups.

At the individual item level (Supplementary Table 1), compared to Non-Latinx White participants, Latinx participants reported significantly higher distrust on Items 5 (“The health care system puts making money above patients’ needs”) and 6 (“The health care system gives excellent medical care”).

### Other variables

Sex and education showed no relationship to either the continuous measure of distrust and its subscales or to the distrust quartiles.

## Discussion

In this first known study evaluating healthcare distrust among an incarcerated population, a moderate positive association was found between age and overall distrust. Respondents’ concerns about the competence of the healthcare system – the ability of the system to provide the expected level of care – drove this association, rather than concerns about discordant values between patients and the healthcare system. Similarly, although race and ethnicity were not significantly associated with overall distrust, competence distrust alone was significantly higher among Non-Latinx White respondents compared to other respondent groups.

The importance of understanding the source of distrust lies in the fact that higher competence distrust has been linked to decreased healthcare-seeking behaviors in prior research. In a study of non-incarcerated women, Yang et al. found that women with higher levels of competence distrust are less likely to have diagnostic tests that require adept physician skill, such as Pap smears for cervical cancer detection (Yang et al., 2011), since they may believe that the physician or the ensuing diagnostic processes are ineffective. For prostate cancer screening, only high values distrust was associated with fewer screenings. However, the study was conducted in a population that generally believed that the medical techniques of the health system were trustworthy (Yang et al., 2013), highlighting the importance of competence trust.

The weak but positive association between healthcare distrust and age was driven by higher distrust among respondents in the 33 to 42-year-old age group, a finding which accords with studies of distrust in the general population (Durant, Legedza, Marcantonio, Freeman, & Landon, 2011; Shenolikar, Balkrishnan, & Hall, 2004). A number of possible factors could contribute to higher distrust among the older two age groups of prisoners. First, the need for healthcare is more likely to arise as one ages, suggesting that younger-aged participants may hold relatively neutral perceptions of trust because they have had fewer healthcare concerns. Older prisoners are more likely to have begun experiencing chronic health conditions and to have experienced them for a longer period than aged-matched individuals in the community (Binswanger, Blatchford, Lindsay, & Stern, 2011). Moreover, they may have faced longer cumulative periods of incarceration, thereby deriving a larger portion of their healthcare needs while in correctional settings. Given that prisoners often perceive the quality of correctional healthcare (at least for select health needs) to be inferior to that available in the community (Christopher et al., 2017), it should not be surprising that older participants distrust the competence of the healthcare available to them.

Ensuring that middle-age to older-age adults who are incarcerated receive healthcare is important for several reasons. First, as noted above, they have greater health needs than their younger counterparts (particularly prisoners > 55) (Greene, Ahalt, Stijacic-Cenzer, Metzger, & Williams, 2018), suggesting that longer delays in receipt of healthcare may contribute to adverse outcomes. They are also likely to be closer to release (Sabol, 2008) and, upon release, face greater mortality compared to community dwelling individuals of comparable age (Binswanger et al., 2011). Still, further research is needed to understand why distrust seems to peak in the second-oldest age quintile, a finding which aligns with some studies in the general population (Armstrong et al., 2006; Born et al., 2009).

The fact that Non-Latinx White respondents held higher levels of competence-related distrust is surprising, particularly in light of data that overwhelmingly shows racial and ethnic minorities have more negative experiences with healthcare (Bulatao, 2004) and higher levels of distrust (Armstrong et al., 2008; Armstrong et al., 2013; Carpenter et al., 2009; Corbie-Smith, Thomas, & St George, 2002; Durant et al., 2011; Gordon, Street Jr., Sharf, Kelly, & Souchek, 2006; Halbert, Armstrong, Gandy Jr., & Shaker, 2006; LaVeist et al., 2009; Rajakumar, Thomas, Musa, Almario, & Garza, 2009). Given these strong and consistent associations found in the general population regarding increased distrust among BIPOC groups, in the absence of further replication the findings from the present study should be interpreted cautiously. If this finding proves valid after subsequent inquiry, it may have more to do with how these different racial and ethnic groups experience healthcare in and out of correctional settings. For example, it may be that Non-Latinx White individuals have had more positive healthcare experiences in the community and therefore, once incarcerated, express greater levels of distrust in the competence of correctional healthcare providers. Conversely, Latinx and Non-Latinx/Non-White individuals may already hold higher levels of distrust based on their past negative healthcare experiences in the community. Assuming these suppositions are true, the higher level of distrust identified here among Non-Latinx Whites may reflect, in part, the underlying institutional disparities in care to which BIPOC groups are already subjected, irrespective of whether they are incarcerated or not.

Another possible and related explanation is that Latinx and Non-Latinx/Non-White individuals experience correctional-based healthcare more positively because they have more equitable access to providers than they do in the community. In other words, care may be more readily available because at least some of the structural obstacles to receiving treatment (e.g., finances, insurance status, transportation) are mitigated by virtue of being incarcerated. Indeed, in the correctional setting in which the present study data were collected, a number of robust public health programs have been launched in recent years to target health needs among prisoners, such as Hepatitis C treatment and colorectal and breast cancer screening programs (Ghidei, Ramos, Brousseau, & Clarke, 2018). These efforts have enabled many people to access these services for the first time, which presumably would contribute to a certain degree of healthcare trust. If so, these programs not only help improve health but could help remedy the social inequality people have experienced. It should be noted, however, that future work should include assessing experiences about healthcare delivery inside versus outside the carceral system and history of healthcare encounters, as these data may shed further light on the paradoxical nature of these findings. Irrespective of these associations, however, prison should not be considered a “healthier” option than living freely, even if some people are accessing healthcare for the first time. Rather than being a positive reflection on the medical care available in the criminal justice system, such access more likely underscores the broader malfunction of the healthcare system outside of prison and the social systems that affect health, insofar as they have failed to provide opportunities for a healthy life in the community that all individuals deserve.

Undoubtedly, addressing health distrust among individuals in correctional settings is a formidable task. Notwithstanding the findings that select groups may have more distrust than others, given the number and complexity of factors that give rise to such distrust, it would be naïve to presume that distrust can be effectively ameliorated by educational interventions or policies aimed at these individual patient groups. Because healthcare distrust is rooted in structural racism and other broad social injustices that affect health, in the absence of strong policies to address these problems, distrust seems unlikely to change.

It would be disingenuous and beyond the scope of the present paper to provide comprehensive recommendations for addressing these critically important problems. At a minimum, however, such efforts to improve distrust need to begin at system, rather than individual-patient, levels. For example, medical schools and healthcare institutions should implement (as some now do) anti-racism training, with focus on lifelong commitment to continually learn how to fight against unjust policies (Bailey et al., 2017) and to make anti-racist choices in their professional and personal lives (Kendi, 2019). These same policies must be extended to all healthcare personnel and trainees that work in criminal justice settings.

### Limitations

Data for this study is drawn from a relatively small sample derived from a single state correctional system, and from a subset of individuals already participating in clinical research while incarcerated; these factors all limit the generalizability of these findings. Most significantly, there were not data collected on past healthcare utilization, either within or without the correctional system, rendering conclusions speculative. While a widely used tool validated across a number of community populations was used to measure healthcare system distrust, the measure has not been specifically validated for use among an incarcerated population. This is an important limitation considering that individuals may be understandably hesitant to express negative views about any aspect of the care they receive while incarcerated. Future research on healthcare distrust among incarcerated individuals would benefit from the development of a validated instrument for measuring healthcare distrust specifically among this population and from examining a wider range of contributing factors including the length of prison stay for respondents, prior access to healthcare inside and outside carceral settings, and perceived quality of these interactions. Future studies should include qualitative interviews with survey respondents to identify specific interventions to reduce distrust and combat inequity, with follow-up to determine the effectiveness of policy changes, such as required anti-racism training and reprioritizing the “health” and “care” aspects of prison healthcare.

## Conclusions

In this novel, exploratory study, overall distrust and competence-related distrust in the healthcare system among incarcerated individuals was significantly higher among 33 to 42-year-olds compared to other age groups. Surprisingly, highest competence-related distrust was reported by Non-Latinx White respondents compared to other racial/ethnic groups, a finding which requires further exploration. These preliminary findings highlight problems that exist among select groups of prisoners in establishing the requisite trust in the healthcare system to address the myriad health problems that prisoners face. Identifying factors related to healthcare system distrust could improve circumstances for individuals currently experiencing incarceration. Addressing healthcare system distrust requires confronting the root issues that give rise to distrust: unjust laws and structural inequities.

## Supplementary Information

**Additional file 1 **: **Supplementary Table 1**. Responses to Individual Items of the Health Care System Distrust Scale. **Supplementary Figure 1**. Distribution of Likert Responses for the (a) Competence and (b) Values subscales of the Health Care System Distrust Scale. Question text can be found in Table 2 in the main document text.

## Data Availability

The datasets analyzed during the current study available from the corresponding author on reasonable request.
