# Supplementary material for: Do prisoners trust the healthcare system?
Source: Health Justice. 2021 Jul 3;9:15. doi: 10.1186/s40352-021-00141-x (PMC8254986; doi:10.1186/s40352-021-00141-x)
Supplement: Supplementary file 1 — Additional file 1 : Supplementary Table 1. Responses to Individual Items of the Health Care System Distrust Scale. Supplementary Figure 1. Distribution of Likert Responses for the (a) Competence and (b) Values subscales of the Health Care System Distrust Scale. Question text can be found in Table 2 in the main document text. [file 40352_2021_141_MOESM1_ESM.docx]

**Supplementary Table 1. Responses to Individual Items of the Health Care System Distrust Scale**

|  | Item # | 1 | | 2 | | 3 | | 4 | | 5 | | 6 | | 7 | | 8 | | 9 | |
| --- | --- | --- | --- | --- | --- | --- | --- | --- | --- | --- | --- | --- | --- | --- | --- | --- | --- | --- | --- |
|  |  | Competence | | Values | | Competence | | Competence | | Values | | Competence | | Values | | Values | | Values | |
|  | n (%) | Mean | p | Mean | p | Mean | p | Mean | p | Mean | p | Mean | p | Mean | p | Mean | p | Mean | p |
| **Age (years)** |  |  |  |  |  |  |  |  |  |  |  |  |  |  |  |  |  |  |  |
| 19-23 | 40 (20) | 2.18 | 0.07 | 2.83 | 0.07 | 2.70 | 0.01 | 2.50 | 0.08 | 2.83 | 0.08 | 2.70 | 0.04 | 2.28 | 0.01 | 2.48 | 0.00 | 2.48 | 0.15 |
| 24-27 | 40 (20) | 2.40 |  | 2.63 |  | 2.55 |  | 2.33 |  | 2.50 |  | 2.43 |  | 2.25 |  | 2.10 |  | 2.15 |  |
| 28-32 | 40 (20) | 2.13 |  | 2.98 |  | 2.60 |  | 2.60 |  | 3.05 |  | 2.60 |  | 2.38 |  | 2.40 |  | 2.33 |  |
| 33-42 | 40 (20) | 2.78 |  | 2.80 |  | 3.28 | * p < 0.02 compared to all quintiles | 2.85 |  | 3.25 |  | 3.13 | * p < 0.03 vs age quintiles 2 and 3 | 2.85 | * p < 0.03 vs age quintiles 2 and 3 | 3.05 | * p<0.04 compared to all other quintiles | 2.58 |  |
| 43-66 | 40 (20) | 2.53 |  | 2.73 |  | 2.68 |  | 2.78 |  | 2.88 |  | 2.80 |  | 2.60 |  | 2.55 |  | 2.08 |  |
| Race/  Ethnicity |  |  |  |  |  |  |  |  |  |  |  |  |  |  |  |  |  |  |  |
| Non-Latinx White | 99 (49.5) | 2.58 | 0.09 | 2.79 | 0.92 | 2.90 | 0.06 | 2.69 | 0.23 | 3.03 | 0.01 | 2.76 | 0.01 | 2.51 | 0.60 | 2.56 | 0.06 | 2.24 | 0.08 |
| Non-Latinx/ non-White | 54 (27) | 2.24 |  | 2.83 |  | 2.48 |  | 2.43 |  | 2.48 | *p<0.01 vs NLW and L | 2.43 | *p<0.004 vs and L | 2.33 |  | 2.24 |  | 2.20 |  |
| Latinx | 47 (23.5) | 2.21 |  | 2.74 |  | 2.78 |  | 2.66 |  | 3.11 |  | 3.02 |  | 2.55 |  | 2.74 |  | 2.62 |  |

**Supplementary Figure 1. Distribution of Likert Responses for the (a) Competence and (b) Values subscales of the Health Care System Distrust Scale.** Question text can be found in Table 2 in the main document text.


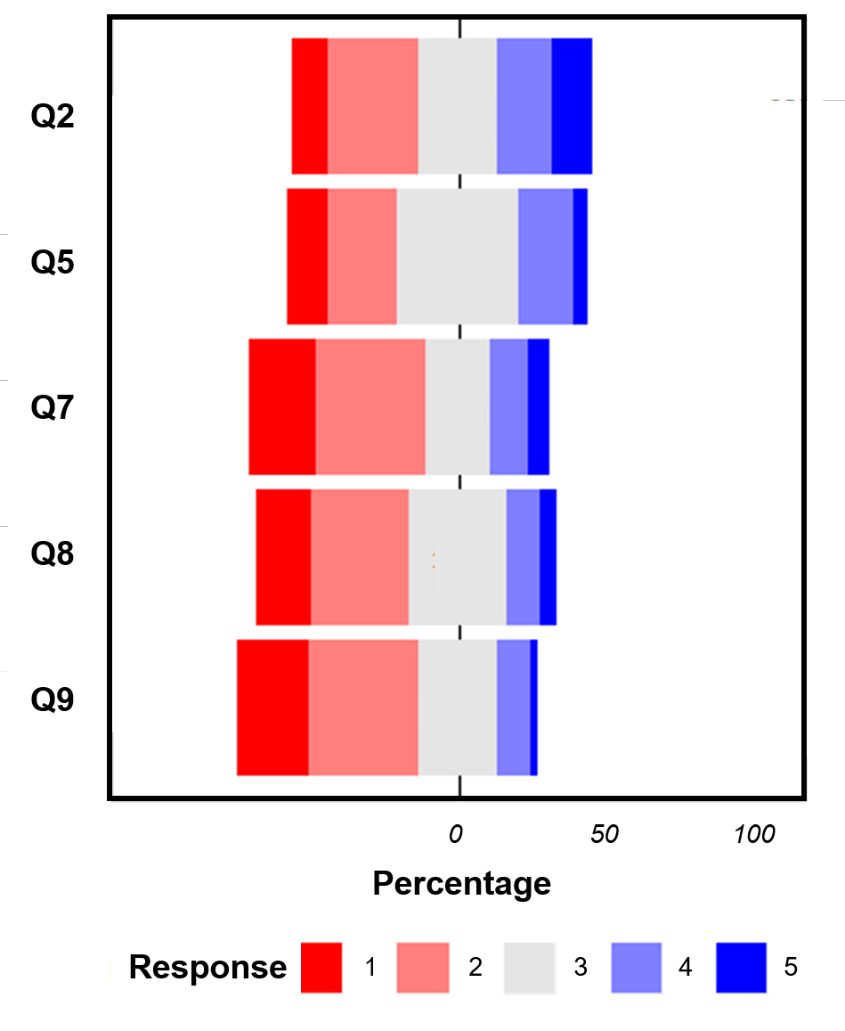

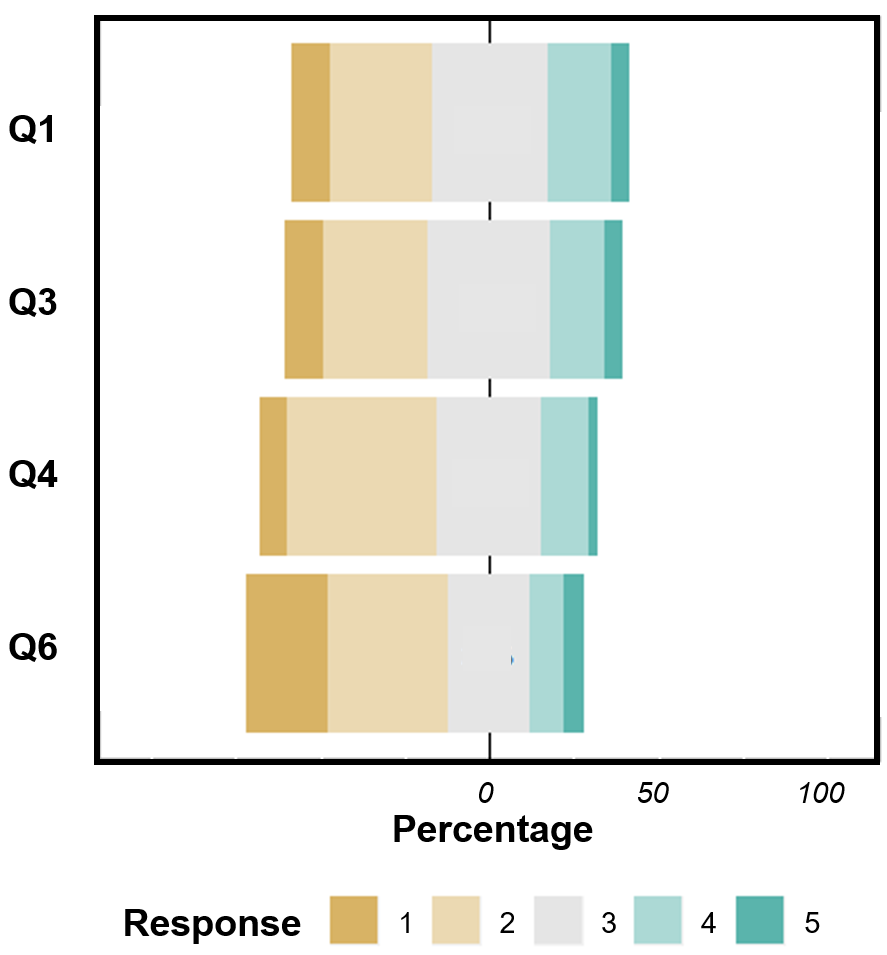


**(b)**

**(a)**
